# Long-term exposure to laboratory conditions: can adaptation reshape the biology of pathogens?

**DOI:** 10.3389/fpls.2026.1972409

**Published:** 2026-10-23

**Authors:** Shailja Singh, Christina Dixelius

**Affiliations:** Department of Plant Biology, Uppsala BioCenter, Swedish University of Agricultural Sciences, Uppsala, Sweden

**Keywords:** cell wall, culture history, infection biology, laboratory adaptation, *Phytophthora infestans*, repeated subculturing

## Introduction

Experimental plant pathology relies heavily on *in vitro* subculturing of bacteria and filamentous pathogens to study their biology and interactions with host plants (Judelson et al., 2009; Kim et al., 2017). Strains are frequently exchanged between research groups and extension services worldwide, and many pathogens of experimental importance are maintained through repeated propagation on artificial media. Although long-term storage at −70 °C or lower can reduce the need for subculturing in some species, cryopreservation is not feasible or reliably effective for all pathogens, leaving continued maintenance in culture as the only practical option in some cases. Among 414 oomycete strains evaluated in one preservation study, 264 (64%) remained viable after one year of storage in liquid nitrogen vapor (Nagai et al., 2005). This indicates that cryopreservation may not provide reliable long-term recovery for all strains. Preservation methods that minimize storage-associated alterations have been developed. However, many microbes still lack efficient long-term preservation methods (Prakash et al., 2020). Importantly, maintaining viability alone is insufficient; preserving wild-type biological functionality is equally critical.

In this Opinion article, we argue that extended cultivation on synthetic media under controlled conditions may act as an overlooked selective environment that reshapes pathogen biology. We focus on how successive subculturing on artificial media can alter infection-related phenotypes and gene expression. Throughout, we distinguish reversible responses to the current culture environment, persistent regulatory states, and heritable genomic changes because these processes are frequently conflated. Examples spanning several taxonomically diverse pathogen groups are summarized in **Table 1**. We further discuss how nutrient sensing and cell wall plasticity might mechanistically link laboratory conditions to infection biology and propose that strain history and subculturing practices should be reported as standard experimental variables. We conclude with recommendations for the pathology research community to improve reproducibility and interpretation of infection assays.

**Table 1:** Examples of phenotypic, transcriptional and virulence-associated changes observed following repeated subculture on artificial media across taxonomically diverse pathogen groups.

Organism	Phylum	Passage / culture conditions	Key phenotypic / virulence outcome^1^	Proposed mechanism^2^	Reversible?^3^	Reference
Oomycota - plant pathogens^4^						
*Phytophthora infestans* 88069	Oomycota	>25 years continuous subculture on rye agar	Swollen germ tube tips; altered infection-structure morphology; infection still proceeds	Unknown; strain background and culture history both candidate factors	Not tested	Singh et al., 2026 (our example)
*P. infestans* (US-23 field isolates)	Oomycota	Sporangia from agar plate cultures (2 to more than 20 transfers) compared with sporangia from tomato leaflet lesions	Much lower indirect germination and aggressiveness of plate sporangia, including after only two agar transfers; 5,398 genes (~34% of expressed genes) differentially expressed, more in the isolate with more transfers (4,791 versus 2,155 genes); one isolate no longer produced lesions after 20 or more transfers	Down-regulated flagellar genes and up-regulated elicitin, nutrient-transporter and stress-response genes in plate sporangia; the authors suggest a progressive, possibly epigenetic process	Yes - restored by host passage	Fry et al., 2019
*P. infestans* (sporangia comparison)	Oomycota	Artificial media compared with plant lesions; sporangium maturity standardized	No significant difference in infection rate, aggressiveness or germination when maturity is standardized; subtle transcriptional differences detected	Sporangium maturity is a key confounding variable; transcriptional differences do not translate into functional impairment under standardized conditions	Not applicable	Leesutthiphonchai and Judelson, 2019
Oomycota - non-plant pathogen						
*Saprolegnia parasitica*	Oomycota (fish pathogen)	Successive *in vitro* passages	Reduced virulence in Atlantic salmon parr; strain-dependent rate of attenuation	Unknown; first documented virulence attenuation by subculture in an oomycete	Yes - restored by host passage	Songe et al., 2014
Ascomycota - plant pathogens						
*Botrytis cinerea*	Ascomycota	8 months, monthly subculture on artificial media	Progressive virulence loss on *Arabidopsis*; no significant genomic mutations detected	Reversible changes in global DNA methylation	Yes - restored after host inoculation	Breen et al., 2022
*Fusarium graminearum*	Ascomycota	50 consecutive weekly subcultures on PDA (SC1, SC23, SC50)	Progressive virulence decline on wheat; reduced conidiation; 1,024 genes differed in methylation between SC50 and the reinfected line (SC50×3)	Differential DNA methylation of genes previously implicated in virulence	Yes - fully restored after three re- infections on wheat	Tini et al., 2021
*Fusarium oxysporum* f. sp. *niveum*	Ascomycota	Successive subculture; aged mycelia increased the incidence of degeneration	Sectoring and degenerate variant formation; reduced cellulase, pectinase and xylanase activities; reduced virulence in soil-inoculation assay but not in root-dipping assay	Downregulation of CWDE genes and several virulence-related transcripts	Not tested	Chang et al., 2020
*Fusarium oxysporum* f. sp. *lycopersici* 4287	Ascomycota	10 serial passages on axenic media across multiple culture conditions; five independent replicate lines per condition	Increased fitness under the selection condition; almost all plate-adapted lines showed reduced invasive growth and virulence on tomato and *Galleria mellonella*, whereas two lines (one on complete and one on minimal medium) had slightly increased virulence on plants^5^	Transposable-element insertions accounted for more than 70% of variants; recurrent adaptive mutations in accessory and core genes	Not tested	López-Díaz et al., 2025
*Magnaporthe oryzae*	Ascomycota	Weekly *in vitro* transfer on artificial media; whole-genome sequencing at multiple time points	Mutations accumulated rapidly before visible phenotypic changes; phenotype largely stable	Accumulation of genomic mutations driven by oxidative damage and UV; genome buffering limits phenotypic expression	Not applicable (virulence not directly tested)	Jeon et al., 2013
Bacteria - plant pathogen						
*Ralstonia solanacearum*	Pseudomonadota	50 consecutive subcultures in SP broth (24 h per passage); three strains of differing initial pathogenicity	Virulent and intermediate strains both became avirulent (0% tomato disease at 21 days after 50 passages, compared with 100% and 29.2% at passage 1); attenuation index increased significantly; the initially avirulent strain was unchanged	Total methylation increased by 7.8% and 38.2% relative to passage 1 (full methylation by 141% and 179%); upregulation of *dam* and *dcm* (methyltransferases) and *ftsZ* (cell-division gene treated as methylase-related in the study) and downregulation of the demethylase gene *alkB*	Not tested	Zheng et al., 2024
Ascomycota - entomopathogenic fungi						
*Metarhizium anisopliae*	Ascomycota (entomopathogen)	Repeated *in vitro* subculture on artificial media	Spore surface adhesion, hydrophobicity and spore-bound Pr1 protease activity all declined after the first subculture; virulence correlated with Pr1 decline	Repression of spore-bound Pr1 serine protease; Pr1 activity proposed as a quality-control marker	Not tested	Shah et al., 2007
*Metarhizium* *anisopliae* and *M. brunneum*	Ascomycota (entomopathogen)	12 successive subcultures on Sabouraud dextrose agar or broken rice; two Strains of *M. anisopliae* and one of *M. brunneum*	No virulence decline against *Tenebrio molitor*; conidial yield was lowest at the twelfth subculture in two strains;	Counterexample: subculture effects are isolate-dependent; species or genus identity alone does not predict the outcome	Not applicable (stable)	Ansari and Butt, 2011
			Spore-bound Pr1 activity fell after the first subculture on agar but rose on rice^5^			
*Beauveria bassiana*	Ascomycota	Continuous subculture on rice grains (20 passages) and PDA (12 passages)	60–68% virulence reduction (12 passages on PDA, 20 on rice) and 49–55% conidial yield reduction after 12 passages; increased oxidative stress, autophagy and apoptosis proteins; decreased DNA repair, energy metabolism and virulence proteins	TOR signaling possibly associated with subculture-induced aging; ROS accumulation; spore-bound Pr1 decline	Yes - restored by passage through *Tenebrio molitor* or *Spodoptera exigua*	Jirakkakul et al., 2018; Safavi, 2011
Ascomycota - yeasts and opportunistic human fungi						
*Saccharomyces cerevisiae* (laboratory strains S288C, W303)	Ascomycota	Decades of laboratory maintenance and repeated bottlenecks since the 1950s	Loss of filamentous growth in many laboratory strains; approximately 9,500 sequence variants between W303 and S288C, affecting around 700 protein-coding genes; elevated protein evolutionary rate relative to a wild strain	Relaxed selection under recurrent laboratory bottlenecks; a single point mutation in *FLO8* accounts for loss of filamentous growth	Not applicable	Liu et al., 1996; Gu et al., 2005; Matheson et al., 2017
*Candida albicans* (clinical isolates)	Ascomycota	10 sequential daily passages on Sabouraud dextrose agar (19 clinical isolates)	Isolate-dependent switching of colony morphology and color; proteinase activity varied significantly with passage, whereas phospholipase and esterase activities were more stable; fluconazole susceptibility changed in both directions (two resistant isolates became susceptible, three susceptible isolates became resistant)	The authors suggest dynamic and reversible regulatory changes	Not tested	Nouraei et al., 2026
*Aspergillus fumigatus*	Ascomycota (opportunistic human pathogen)	25 passages over 231 days on *Galleria* extract agar	Reduced virulence; increased antifungal tolerance	Proteomic reprogramming, with oxidative stress response proteins most altered; culture substrate shapes the direction of change	Not tested	Curtis et al., 2023
Basidiomycota - edible mushroom (non-pathogen)						
*Volvariella volvacea*	Basidiomycota	20 months continuous mycelial subculture (every 3 days); up to 19 tissue-isolation successions	Progressive degeneration: reduced hyphal growth, biomass and lignocellulase activity; loss of antioxidant	ROS accumulation, leading to decreased antioxidant enzyme activity, mitochondrial impairment and cellular senescence	Not tested	Zhao et al., 2022; Zhao et al., 2023

Bacteria - animal and human pathogens						
*Mycobacterium bovis*	Actinomycetota	230 serial passages *in vitro* (deliberate creation of the BCG vaccine strain)	Complete attenuation of virulence in humans and animals	Deletion of the RD1 genomic region (Mahairas et al., 1996); the ESAT-6 protein (encoded by *esxA*) was also absent from all BCG substrains tested (Harboe et al., 1996)	Not applicable (deliberate vaccine attenuation)	Mahairas et al., 1996; Harboe et al., 1996
*Burkholderia pseudomallei*	Pseudomonadota	Serial passage in LB broth (up to 28 passages)	Increased invasion, intracellular replication, plaque formation and *Galleria* *mellonella* killing^5^	Proteomic reprogramming; altered LPS profiles; passage-induced change is not always attenuating	Not tested	Duangurai et al., 2020
*Xenorhabdus nematophila*	Pseudomonadota	Serial passage selecting for fast growth in artificial broth (80 independent lines)	Shortened lag phase; increased virulence in insects in selected lineages^5^	*flhDC* regulon evolved rapidly; the link between fast growth and virulence remains multifactorial	Not tested	Chapuis et al., 2011
Gram-positive and Gram-negative fish pathogens (*Lactococcus lactis*, *Escherichia coli* and others)	Bacillota; Pseudomonadota	56 consecutive subcultures in tryptic soy broth	Gram-positive pathogens showed progressive virulence loss; Gram-negative pathogens maintained higher virulence but with gradual decline; no genetic change detected in AMR genes	Phenotypic rather than genetic change; Gram- positive pathogens more susceptible to attenuation under non-selective conditions	Not tested	Bag et al., 2025
Viruses						
Porcine deltacoronavirus	Pisuviricota	240 passages in LLC-PK cell culture	Complete loss of pathogenicity in neonatal piglets; altered acid stability and S protein receptor binding	Structural mutations in the Spike (S) protein and nsp3	Not applicable (vaccine use)	Zhang et al., 2024
Vaccinia virus (Lister strain)	Nucleocytoviricota	Serial passage in chick embryo fibroblasts	Loss of tissue dissemination; temperature sensitivity; small plaque phenotype (the LC16m8 attenuated smallpox vaccine strain)	Frame-shift mutation truncating the *B5R* envelope protein gene; loss of extracellular enveloped virion formation	Not applicable (vaccine use)	Morikawa et al., 2005
Influenza B virus	Negarnaviricota	Selection of high-growth variants by serial passage in embryonated chicken eggs	Three amino acid substitutions near the HA receptor-binding pocket changed glycan binding; variants with G141E bound almost exclusively α2,3-linked sialylated glycans	Amino acid substitutions in the HA receptor-binding pocket selected by egg adaptation	Not applicable	Lugovtsev et al., 2009
Protists						
*Leishmania donovani*	Euglenozoa (kinetoplastid)	Long-term *in vitro* promastigote culture (early compared with late passage)	Virulence loss; impaired conversion to the virulent metacyclic stage; altered macrophage infection	Chromosome copy number variation; transcriptional dysregulation of ABC transporters and calpain-like cysteine proteases	Not tested	Sinha et al., 2018

*Plasmodium falciparum*	Apicomplexa	Prolonged continuous erythrocytic culture without receptor-selection pressure	Loss of cytoadherence to endothelial receptors; clonal switching of the surface antigenic phenotype at about 2% per generation	Stochastic *var* gene expression switching altering PfEMP1 display; unselected culture enriches non-cytoadherent parasites	Partially - cytoadherence can be re-selected by receptor panning	Roberts et al., 1992; Smith et al., 1995

1 ABC, ATP-binding cassette; AMR, antimicrobial resistance; BCG, bacille Calmette-Guérin; CWDE, cell-wall-degrading enzymes; HA, hemagglutinin; LB, lysogeny broth; LLC-PK, Lilly Laboratories Cell-Porcine Kidney; LPS, lipopolysaccharide; PDA, potato dextrose agar; PfEMP1, *Plasmodium falciparum* erythrocyte membrane protein 1; ROS, reactive oxygen species; SC, subculture number; SP, sucrose peptone; TOR, target of rapamycin; UV, ultraviolet.

2 Specific gene, protein and genomic-region names are given as reported in the cited studies. Genes: *dam*, *dcm*, *ftsZ* and *alkB* (*Ralstonia solanacearum*); *FLO8* (*Saccharomyces cerevisiae*); *flhDC* (*Xenorhabdus nematophila*); *B5R* (vaccinia virus); and *var* (*Plasmodium falciparum*). Proteins: Pr1 (*Metarhizium anisopliae*, *Beauveria bassiana*), ESAT-6 (*Mycobacterium bovis*, encoded by *esxA*), nsp3 (porcine deltacoronavirus) and PfEMP1 (*Plasmodium falciparum*). Genomic regions: RD1 (*Mycobacterium bovis*).

3 Reversible: indicates whether the reported change was shown to revert. Yes: reversal was experimentally demonstrated, in every case here by passage through a host. Partially: the original phenotype could be recovered by selection but not spontaneously. Not tested: reversibility was not examined in the cited study. Not applicable: the question does not arise, either because no change was observed, because attenuation was deliberate (vaccine strains), or because virulence itself was not measured; the reason is given in the cell.

4 Shaded rows indicate section headings used to organize the entries by taxonomic group and pathogenic lifestyle.

5 Passage-induced change is not universally attenuating. *Burkholderia pseudomallei, Xenorhabdus nematophila* and some *Fusarium oxysporum* f. sp. *lycopersici* strains showed increased virulence after *in vitro* passage, and *Metarhizium* isolates showed no decline, indicating that the direction and magnitude of change depend on the organism, isolate, passage regime and assay used.

### Successive culturing on artificial media can influence infection-related traits

Repeated subculturing is known to alter pathogenic traits, including virulence-associated phenotypes, in fungal and bacterial pathogens (Chang et al., 2020; Bag et al., 2025). The effects are not always consistent across assays; successive subculture of *Fusarium oxysporum f.* sp. *niveum* generated degenerate variants with reduced cell-wall-degrading enzyme activity and attenuated virulence in a soil-infestation assay, yet the same variants were indistinguishable from the parental strain using a root-dipping assay (Chang et al., 2020). Oomycetes are no exception: in the fish pathogen *Saprolegnia parasitica*, successive subculturing reduced virulence in a strain-dependent manner, with virulence restored after passage through the host (Songe et al., 2014). Those authors noted that attenuation had not previously been documented in oomycetes, including plant pathogens. *Phytophthora infestans*, the causal agent of late blight disease on potato and tomato, is one of the most studied oomycetes (Nowicki et al., 2012). Sporangia of *P. infestans* produced on artificial media can differ from those produced on host lesions in germination, aggressiveness, and transcriptional profiles, with more extensive transcriptional differences in the isolate with the longer history on agar media (Fry et al., 2019). Transcriptomic comparisons further revealed that culture conditions can alter metabolic and regulatory programs relevant to infection, including elevated levels of stress-response genes and reduced activation of flagellar genes (Fry et al., 2019). Such differences are not universal; when sporangial maturity was standardized, media- and lesion-derived sporangia showed equivalent infection potential and aggressiveness despite subtle transcriptional divergence (Leesutthiphonchai and Judelson, 2019). Experiments using other pathogens demonstrate that repeated cultivation can produce diverse phenotypic and molecular changes. Serial passage of a clonal *Fusarium oxysporum* f. sp. *lycopersici* isolate on axenic media increased fitness under the same culture conditions and generated abundant transposable-element insertions. Most plate-adapted lines showed reduced virulence, although some showed the opposite pattern (López-Díaz et al., 2025). In the bacterial plant pathogen *Ralstonia solanacearum*, 50 consecutive subcultures rendered both a virulent and an intermediate strain avirulent on tomato, whereas an initially avirulent strain was unaffected; the reduction in virulence was accompanied by increased genomic DNA methylation (Zheng et al., 2024). Comparable changes are documented across oomycetes, fungi, bacteria, viruses and protozoa (**Table 1**). These observations raise a broader concern. Are we studying pathogens as they exist in nature, or pathogens whose behavior and phenotypes have been shaped in part by their laboratory history?

### Could laboratory nutrient environments influence infection-associated traits?

Microbes preferentially assimilate favorable sugars before utilizing other available carbon sources, a phenomenon known as carbon catabolite repression (CCR), first characterized in *Saccharomyces cerevisiae* and, among filamentous fungi, studied most extensively in *Aspergillus nidulans* (Adnan et al., 2018). CCR influences numerous functions, including growth and virulence, and in filamentous pathogens this system often involves regulation of regulation of cell-wall-degrading enzymes. Direct evidence comes from filamentous plant pathogens themselves. For example, in *Fusarium oxysporum*, disruption of *FoSNF1* reduced the expression of cell-wall-degrading enzyme genes and attenuated virulence on *Arabidopsis* and cabbage (Ospina-Giraldo et al., 2003). Similarly, disruption of *MoCreC* in *Magnaporthe oryzae* affected vegetative growth, conidiation, appressorium formation and virulence. It also activated cell-wall-degrading enzyme genes and compromised cell wall integrity (Matar et al., 2017). Deletion of *VdCreA* or *VdCreC* in *Verticillium dahliae* altered growth, microsclerotium formation, virulence and the expression of plant cell-wall-degrading enzymes (Wang et al., 2024). Nutrient sensing at the fungal plasma membrane is crucial for adaptation to changing environments, and multiple receptors are involved in these complex processes (Van Dijck et al., 2017). In *Candida albicans*, the choice of carbon source affects cell wall structure and consequently influences virulence (Ene et al., 2012). Whether such culture-induced responses remain reversible or become more persistent during repeated media passages is unknown, but either could leave molecular or phenotypic signatures in strains maintained continuously in culture. Although many mechanistic studies have been performed in fungi, oomycetes also respond strongly to environmental nutrient cues, and similar principles may apply (Judelson et al., 2009). *Phytophthora* species are routinely cultivated on nutrient-rich media such as rye-sucrose agar (Judelson et al., 2009), and they utilize glucose, sucrose and maltose as carbon sources (Khalil and Alsanius, 2009). Whether prolonged growth under such conditions affects pathogen cell wall composition has received little attention.

Cell walls of filamentous plant pathogens are in intimate contact with their host plants and contribute to structural integrity, secretion processes, and the formation of infection structures required for host penetration. The cell wall composition of oomycetes differs fundamentally from that of fungi: *P. infestans* synthesizes walls composed primarily of cellulose and β-glucans rather than the chitin-rich structures characteristic of true fungi (Mélida et al., 2013). The cell wall also has a direct developmental role, since silencing of cellulose synthase genes leaves *P. infestans* unable to generate functional infection structures, indicating that cellulose is required for the developmental program underlying pathogenesis (Grenville-Briggs et al., 2008). These two observations frame our central hypothesis. Cell walls respond dynamically to nutrient and stress signals. Prolonged growth on carbohydrate-rich media may therefore alter wall composition or remodeling, influencing the infection structures formed by the pathogen. This remains untested, since cellulose and β-glucan content has not been compared between freshly isolated and long-term subcultured *Phytophthora* strains. We present this as a potential explanation that remains to be experimentally validated, as no study has yet shown that prolonged cultivation alters cellulose or β-glucan abundance in *P. infestans*. This hypothesis could be tested directly by comparing freshly recovered and serially subcultured isolates using biochemical fractionation and quantitative determination of cellulose and β-glucan content, together with expression analysis of genes involved in cell-wall biosynthesis and remodeling across defined intervals. The question concerns pathogens that can be maintained in culture, whereas obligate biotrophs depend on living host tissue and generally cannot be maintained on artificial media (Kemen and Jones, 2012).

### Atypical infection phenotypes: when laboratory strains challenge interpretation

Atypical infection phenotypes observed in widely used laboratory strains provide concrete examples of how culture history may influence interpretation of experimental results. The *P. infestans* isolate 88069 was collected from tomato in the Netherlands in 1988 (Knapova and Gisi, 2002), is thought to have been maintained in culture ever since, and is now central to many molecular studies. Confocal images of this strain in our recent study show germ tubes with swollen apical tips, a morphology that differs from descriptions of field material, although the infection proceeded as expected (Singh et al., 2026). The origin of this phenotype is unresolved. It could reflect prolonged maintenance in culture, the strain background, or the assay conditions, and we present it as an observation requiring investigation rather than as evidence that laboratory adaptation caused it. Natural populations of *P. infestans* are in any case genetically and phenotypically variable (Wang et al., 2025; Zhang et al., 2026), and unreported differences in maintenance practice add a further level of variation. These strain-to-strain differences do not imply that laboratory strains are “abnormal” or unsuitable for molecular studies. Rather, they highlight the need to consider strain history and experimental context when interpreting infection-associated phenotypes.

## Discussion

The central challenge is not whether laboratory strains are useful, since they are indispensable, but whether their biological history is considered when experimental results are interpreted. Repeated propagation keeps a strain alive, but it also exposes it to an artificial environment where selection can favor adaptation to culture. Traits that improve growth, sporulation, or survival in culture may then become enriched, even when they give no advantage during infection of the host.

These culture-associated changes can occur at three levels. The first is a reversible physiological or transcriptional response to the current culture environment. The second is a persistent regulatory state, including an epigenetic state, that may survive transfer to a new environment or host. The third is a heritable genomic change, such as a mutation, structural variation, or a transposable-element insertion, that can persist across generations and is harder to reverse. Observations across several pathogens illustrate these levels. In *Magnaporthe oryzae*, mutations accumulated during subculture prior to any visible change in phenotype (Jeon et al., 2013). In *Fusarium graminearum*, subculturing lowered virulence and host passage restored it, and the change was accompanied by differences in DNA methylation (Tini et al., 2021). In *Botrytis cinerea*, eight months of culture reduced virulence, which recovered after host passage; the change was accompanied by substantial changes in DNA methylation, and the authors identified no mutations they considered sufficient to explain it (Breen et al., 2022). No minimally passaged field strain of isolate 88069 is currently available, which limits how directly these questions can be addressed in *P. infestans*.

Laboratory-maintained strains are also often preferred for transformation and other molecular work, because established protocols exist for them. Taken together, the cases in **Table 1** do not point to a single mechanism of laboratory adaptation, and the outcomes are not always a loss of virulence, but they do show that culture history deserves consideration as an experimental variable. Addressing this question in *P. infestans* would require a dedicated experiment. A minimally passaged or freshly recovered isolate would be propagated independently in separate cultures, with defined intervals and material archived throughout. Phenotypes, virulence, transcriptomes, genomes, methylomes and small-RNA profiles could then be assessed over time, and host passage used to test reversibility. Transcriptome data alone cannot determine whether changes in gene expression are associated with DNA methylation or small RNAs, making methylome and small-RNA profiling important complements. Such a design would help distinguish the three levels of change and identify when divergence first becomes detectable.

## Conclusion

Reproducibility is a persistent concern in experimental plant pathology. We therefore propose that authors report key aspects of a pathogen’s maintenance history, including strain origin, year of isolation where known, history on artificial media, storage conditions, and the propagation regime used immediately before experiments. Such reporting requires minimal additional effort but could improve interpretation of infection assays, resistance screens, and comparisons between laboratory-maintained and field isolates. In parallel, improved long-term storage strategies are needed to preserve pathogens while maintaining both viability and wild-type functionality.
